# Charge transport mechanism in the forming-free memristor based on silicon nitride

**DOI:** 10.1038/s41598-021-82159-7

**Published:** 2021-01-28

**Authors:** Andrei A. Gismatulin, Gennadiy N. Kamaev, Vladimir N. Kruchinin, Vladimir A. Gritsenko, Oleg M. Orlov, Albert Chin

**Affiliations:** 1 Rzhanov Institute of Semiconductor Physics. Siberian Branch, Russian Academy of Sciences, Novosibirsk, Russia; 2 Novosibirsk State University, 2 Pirogov Street, Novosobirsk, Russia 630090; 3 Novosibirsk State Technical University, 20 K. Marx Ave., Novosibirsk, Russia 630073; 4 Molecular Electronics Research Institute, 6/1 Academician Valiev Street, Zelenograd, Moscow Russia 124460; 5 Moscow Institute of Physics and Technology, 9 Institutskiy Per, Dolgoprudny, Moscow Region Russia 141701; 6 Department of Electronics Engineering, National Chiao Tung University, Hsinchu, 300 Taiwan

**Keywords:** Materials for devices, Nanoscale devices, Applied physics

## Abstract

Nonstoichiometric silicon nitride SiN_*x*_ is a promising material for developing a new generation of high-speed, reliable flash memory device based on the resistive effect. The advantage of silicon nitride over other dielectrics is its compatibility with the silicon technology. In the present work, a silicon nitride-based memristor deposited by the plasma-enhanced chemical vapor deposition method was studied. To develop a memristor based on silicon nitride, it is necessary to understand the charge transport mechanisms in all states. In the present work, it was established that the charge transport in high-resistance states is not described by the Frenkel effect model of Coulomb isolated trap ionization, Hill–Adachi model of overlapping Coulomb potentials, Makram–Ebeid and Lannoo model of multiphonon isolated trap ionization, Nasyrov–Gritsenko model of phonon-assisted tunneling between traps, Shklovskii–Efros percolation model, Schottky model and the thermally assisted tunneling mechanisms. It is established that, in the initial state, low-resistance state, intermediate-resistance state and high-resistance state, the charge transport in the forming-free SiN_*x*_-based memristor is described by the space charge limited current model. The trap parameters responsible for the charge transport in various memristor states are determined.

## Introduction

Amorphous oxide SiO_2_ and silicon nitride Si_3_N_4_ are two key dielectrics in silicon devices. Silicon oxide has a low density of surface states at the interface with silicon and a low trap concentration in the dielectric bulk. Silicon nitride, on the contrary, has high electron and hole trap concentrations^1^. The ability to localize electrons and holes injected into silicon nitride allows one to use it as a storage medium in TaN-Al_2_O_3_-Si_3_N_4_-Si (TANOS) charge trap flash memories^2–4^.

Currently, non-volatile memristor-based memories are intensively developed. A memristor-based Resistive Random-Access Memory (ReRAM) that stores information for 10 years at 85 °C is developed. On the other hand, the memristor imitates the synapse properties, and it opens up prospects for the neuromorphic electronic device development that mimics brain activities^5^.

The memristor effect is the dielectric reversibly changes from a high-resistance to a low-resistance state when a short current pulse is applied. The memristor effect is observed in a wide class of dielectrics, such as Ta_2_O_5_^6^, HfO_2_^7^, ZrO^8^, TiO_2_^9–12^, Al_2_O_3_^13^, Nb_2_O_5_^14^, SiO_*x*_^15–18^, GeO_2_^19^, Si_3_N_4_^20–24^, NiO^25^, perovskites^26,27^, organic films^28^, etc. An important role of oxygen vacancies in the memristor switching was established^29^.

An important issue in the memristor memory is the forming process. In most cases, memristors can only switch after applying a first high voltage pulse (compared to the switching voltage). For example, the forming voltage of a 10 nm thick tantalum oxide memristor is 6 V, while the switching voltage is 1 V^30^. The forming process takes place in the pre breakdown dielectric field, which significantly reduces the memristor reliability due to the possibility of breakdown. In Ref.^31^, to suppress the memristor forming process, it was proposed to use the non-stoichiometric oxide enriched in the metal, in which there is a high oxygen vacancy concentration. There are several ways to achieve high oxygen vacancy concentration: by introducing an active metallic impurity in the oxide layer^32^, adding a non-stoichiometric oxide layer to the stoichiometric layer^33,34^ and applying a thin metal layer with a chemically active metal on the stoichiometric oxide layer^35^.

Silicon nitride is widely used in the silicon technology. The physical properties of non-stoichiometric silicon nitride vary over a wide range with a change in the chemical composition. Thus, the non-stoichiometric SiN_*x*_ bandgap varies in the range from 1.6 eV (amorphous Si^36^) to 4.5 eV (amorphous Si_3_N_4_^37^).

The memristor leakage currents in the high and low-resistance states set the power necessary for the memristor switching. Its power consumption needs to be reduced, especially in devices for mobile applications. The leakage current is determined by the charge transport mechanism in the memristor. Therefore, the study of the charge transport mechanism in the memristor is an urgent and important task.

Charge transport mechanisms can depend on the technology for producing silicon nitride in a memristor. The charge transport in silicon nitride is described by the multiphonon mechanism of isolated trap ionization with the energy of *W*_t_ = 1.6 eV^1,38^. The charge transport in the memristor based on silicon nitride in the low-resistance state was interpreted in terms of the Schottky effect^39,40^. However, similar results were interpreted based on the Frenkel effect^20,24,39^. The phonon-assisted tunneling between traps was used to explain the charge transport in Ref.^23^. In Refs.^22,41^, the memristor charge transport is interpreted based on the model of space charge limited currents.

Thus, at present, various mechanisms are assumed in the charge transport interpretation in SiN_*x*_-based memristors. Perhaps, this is due to the fact that charge transport mechanisms depend on the silicon nitride fabrication technology in the memristor structure.

The aim of this work is to study the charge transport mechanism of a SiN_*x*_-based memristor synthesized by the Plasma-Enhanced Chemical Vapor Deposition (PECVD) method. To unambiguously establish the charge transport mechanism, the memristor current–voltage characteristics were measured at different temperatures in all states. We have chosen the temperature measurement range 300–400 K. At a low temperature, the conductivity is no longer determined by the thermal generation of free charge carriers, and the charge transport at a low voltage can be different, but, at a high voltage, the main charge transport mechanism remains the same^42^. We assume that this temperature range is enough to find the main charge transport mechanism.

## Results

**Figure 1:**
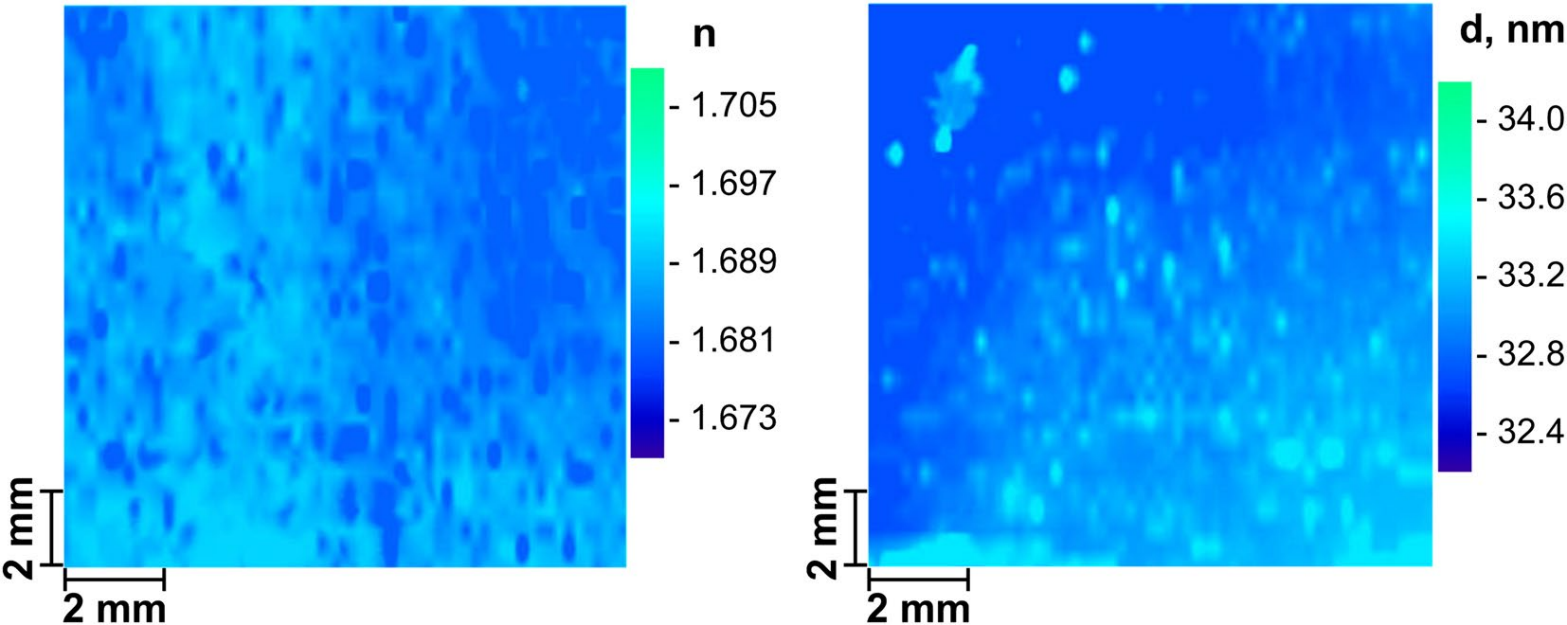
Ellipsometric mapping of refractive index *n* and thickness *d* of p^+^-Si/SiN_x_/Ni memristor structure (hν = 1.96 eV).

The ellipsometric mapping of refractive index *n* and thickness *d* of the p^+^-Si/SiN_x_/Ni memristor structure (at hν = 1.96 eV) synthesized by PECVD is shown in Fig. 1. The SiN_*x*_ dielectric film has the high homogeneity in thickness *d* = 33 nm (~ 2.4%) and refractive index *n* = 1.689 (~ 0.5%).

**Figure 2:**
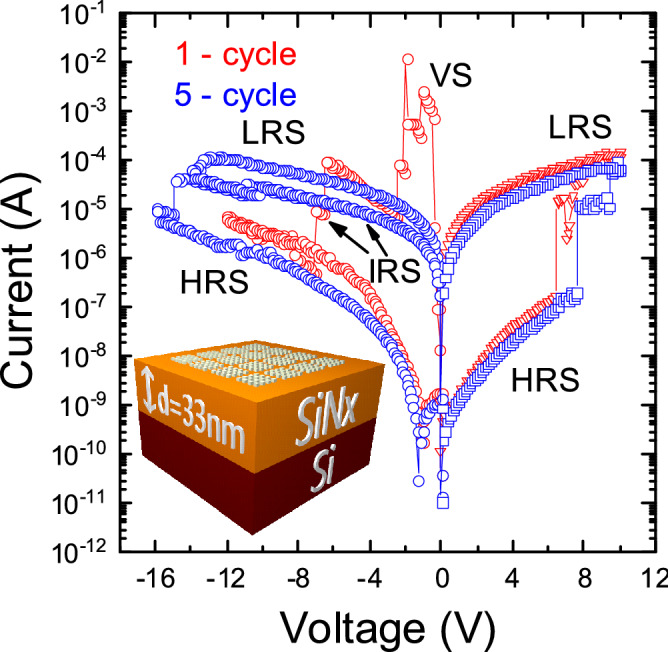
*I–V* characteristics of p^+^-Si/SiN_x_/Ni memristor cycles.

The current–voltage (*I*–*V*) characteristic of the memristor switching cycle at the voltage sweep is shown in Fig. 2. When a negative voltage is applied to the Ni electrode, the memristor structure is immediately in a highly conductive state. At voltage − 1.1 V, the switching from the initial virgin state to the low-resistance state begins. At voltage − 7 V, the memristor is switched to the high-resistance state through the memristor intermediate resistance states. The breakdown voltage of our nitride-based memristors is 19 constant voltage. When the positive voltage of + 7 V is applied to the Ni electrode, the memristor is switched from the high-resistance state to the low-resistance state and at + 10 V it gets the full switching to the LRS (Fig. 2). With the entire switching cycle, four memristor states can be distinguished: initial/virgin State (VS), high-resistance state (HRS), low-resistance state (LRS) and intermediate-resistance state (IRS). After 5 cycles we can see that − 13 V is not enough to switch from the LRS to HRS, we need to apply − 15 V to switch to the HRS. The reset voltage is gradually increasing from one switching cycle to the other and is stopped at 10 cycles at around − 18 V. Typically, the forming process voltage is greater than the memristor set/reset voltage, and the current before the forming process is less than in the HRS. In our case, the forming process voltage is less than the set/reset voltage and the current is greater than in the LRS. Although, in our memristor, there is the process similar to the forming process, this process requires a low voltage, which is an advantage of our memristor. As our memristor does not require a prebreakdown voltage as the forming process in a classical memristor and it needs a less voltage value to switch to the working resistance than set/reset voltage, it can be said that our memristor is forming-free.

The p^+^-Si/SiN_x_/Ni memristor structure is the laboratory structure for study the charge transport mechanism. The 33 nm film was used because it is easier to control the thickness and composition of the obtained SiN_x_ layer by optical methods. Therefore, the SiN_x_-based 33 nm thick memristor does not have many applications as a memristor due to the very high set/reset voltage. But the obtained results can be extrapolated to thinner (4–5 nm) and, thereby, more realistic PECVD memristor structures^24^ and a metal–nitride–oxide–silicon memristor^43,44^.

**Figure 3:**
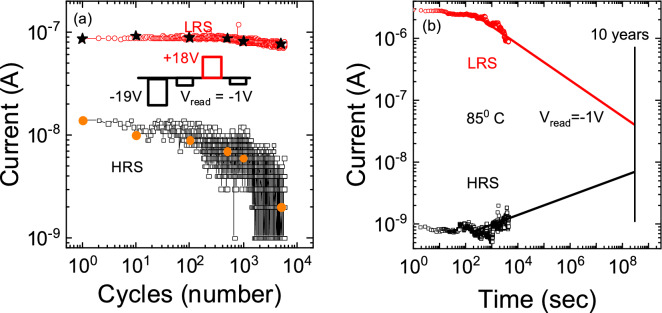
(**a**) Endurance of p^+^-Si/SiN_x_/Ni memristor cycles. (**b**) Retention of p^+^-Si/SiN_x_/Ni memristor cycles at 85 °C.

The typical endurance characteristic was measured by reading the resistance values at the V_read_ = -1 V pulse between 18 V and -19 V pulses which change the resistance state (see Fig. 3a). The memristor endurance with a resistance ratio of about one order holds at least 5000 switching cycles. The memristor memory window was increasing with more cycles. But the HRS became less stable. In Fig. 3b is the memristor retention at 85 °C in the HRS and LRS. The experimental data approximation on the retention in the LRS and HRS by 10 years was carried out. By 10 years, the ratio of current in the LRS to the ratio of current in the HRS at − 1 V is about one order. The endurance and retention measurements allow us to show that this SiN_*x*_-based structure not only exhibits the memristor properties but also preserves them over time. There is a difference in the LRS in Fig. 3a and b. This difference is mainly due to the fact that the LRS resistance was measured in the pulse mode for Fig. 3a and in the DC mode for Fig. 3b. This difference is typical of memristors and was observed in numerous reports^45–47^. Most likely, the resistive switching from the HRS to LRS occurs not fully in the pulse mode.

**Figure 4:**
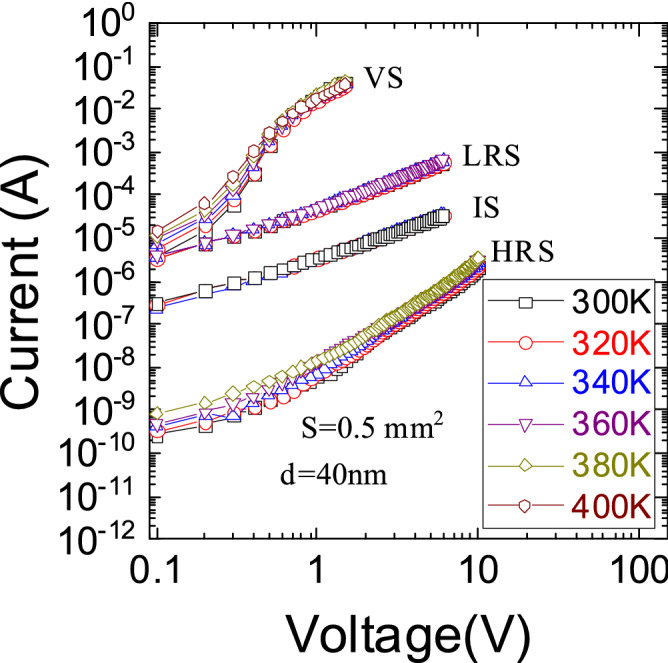
*I–V* characteristics of p^+^-Si/SiN_x_/Ni in VS, IRS, LRS and HRS at different temperatures in a double logarithmic scale.

To identify the charge transport mechanism, the current temperature dependences in all states were measured (Fig. 4). The current temperature dependence of the VS is low at a low voltage and does not exist at a high voltage. But, at the increasing temperature, the current at a low voltage in the VS is still increasing. The *I*–*V* characteristics in the SiN_*x*_-based memristor in the VS are similar to *I*–*V* characteristics in SiO_*x*_-based memristor in the VS^18^. This mean filament can only be composed of Si or more silicon-rich SiN_*y*_ than SiN_*x*_.

The charge transport in the HRS was analyzed by Schottky effect^48^, thermally assisted tunneling (TAT)^49,50^, Frenkel^51,52^, Hill–Adachi (H–A)^53,54^, Makram-Ebeid and Lannoo (ME–L)^55^, Nasyrov–Gritsenko (N–G)^56^, Shklovskii–Efros (S–E)^57,58^ and the space charge limited current (SCLC)^59–62^. Since other resistance states very weakly depend on temperature at a high voltage values, they could be simulated only by Fowler Nordheim^63^ and SCLC models. The VS has the temperature dependence at a low voltage and does not have the temperature dependence at a high voltage. The charge transport mechanism models do not account for dynamic processes at low voltage values. Therefore the VS could be simulated only by Fowler Nordheim^63^ at a high voltage. The SCLC model can simulate the VS in the whole voltage range because it consists of two different parts.

The simulation parameters by the Schottky effect, TAT, Frenkel, Hill–Adachi, Shklovskii–Efros, Makram-Ebeid and Lannoo and Nasyrov–Gritsenko models of the experimental data of the memristor in the HRS are presented in Table 1.

**Table 1:** Fitting different model parameters for simulating the *I*–*V* characteristics of the SiN_*x*_-based memristor.

State	Schottky effect	TAT	Frenkel effect	H–A	ME–L	N–G	S–E
HRS	*W*_*0*_ = 0.2 eV m* = 0.6 × 10^–12^ *m*_e_ *ε*_*∞*_ = 12.3	*W*_*0*_ = 0.1 eV m* = 6 *m*_e_ *S* = 2 nm^2^	*N* = 0.4 cm^−3^ *W* = 0.23 eV *ν* = 5.6 × 10^13^ s^−1^ *ε* = 50	*N* = 3.5 × 10^21^ cm^−3^ *W* = 0.3 eV *ν* = 10^–52^ s^−1^ *ε* = 2.86	*N* = 4 cm^−3^ m* = 0.5*m*_e_ *W*_t_ = 0.25 eV *W*_opt_ = 0.5 eV	*N* = 3.5 × 10^21^ cm^−3^ m* = 90 *m*_e_ *W*_t_ = 0.3 eV *W*_opt_ = 0.6 eV	*j*_0_ = 3 × 10^–5^ A/cm^2^ *W* = 0.24 eV *V*_0_ = 0.5 eV *a* = 0.8 nm
	Fowler–Nordheim model						
IS	m* = 0.5*m*_e_; *W* = 1.21 eV						
LRS	m* = 0.5*m*_e_; *W* = 0.1.15 eV						
VS	m* = 0.5*m*_e_; *W* = 0.4 eV						

The unphysically small effective mass value m^*^ = 0.6 × 10^–12^ *m*_e_ and large high-frequency dielectric constant value *ε*_*∞*_ = 12.3 (from ellipsometry measurements *ε*_*∞*_ = *n*^2^ = 1.69^2^ = 2.86) obtained from the Schottky model simulation indicate the inapplicability of this model (Table 1) for the HRS. When fitting the experimental HRS data by the TAT model, the large effective mass value m^*^ = 6 *m*_e_ and small potential barrier height at the Ni/SiN_*x*_ interface *W*_0_ = 0.1 eV are obtained. Therefore, the model is not applicable to describing the charge transport in the SiN_*x*_-based memristor in the HRS. The unphysically small trap concentration value *N* = 0.4 cm^−3^ and large high-frequency dielectric permittivity value *ε*_*∞*_ = 50 are obtained from the Frenkel effect model simulation. Thus, the Frenkel effect model does not describe the charge transport in the SiN_*x*_-based memristor in the HRS (Table 1). When simulating at 300 K by the H–A model, reasonable values of the trap concentration, ionization energy and high-frequency dielectric constant are obtained (Table 1). But the H-A model gives an inverse temperature dependence in contrast to the experimental data. Thus, the H-A model does not describe the charge transport in the SiN_*x*_-based memristor in the HRS (Table 1). The simulation by the ME–L model gives the unphysically low trap concentration value *N* = 4 cm^−3^. Thus, the ME–L model does not describe the charge transport in the HRS (Table 1). The simulation by the N-G model yields the anomalously large effective electron mass m^*^ = 90 m_e_. Hence, the N-G model does not describe the charge transport in the SiN_*x*_-based memristor in the HRS (Table 1). The comparison of the experimental data with the S-E percolation model gives the following parameters: *I*_0_ = 3 × 10^–5^ A, *W* = 0.24 eV, *V*_0_ = 0.5 eV and *a* = 0.8 nm. The percolation model does not take into account the possibility of tunneling through the barriers, but, when *a* is 0.8 nm, the classical approximation condition for applying the model does not work. Hence, the S–E model does not describe the charge transport in the SiN_*x*_-based memristor in the HRS (Table 1).

The Fowler–Nordheim model does not describe the charge transport mechanism in the VS, IRS and LRS. The fitting parameters in Table 1 are only in case when the Fowler–Nordheim simulated curve intersects with the experimental data curve from the VS, IRS and LRS.

### Space-charge-limited current model

In the classical case, the SCLC^64,65^ mechanism explains a similar conductivity as can be seen in Fig. 4. The classical SCLC model does not explain the bipolar switching and the change in the size of the filament from resistance to resistance. Currently, the charge transport analysis in the memristor was carried out by the SCLC model but without an in-depth analysis of the model parameters^66–68^. To account for the differences in memristors from the classical SCLC model, in different resistance states the filament size is changed. With these assumptions, the *I-V* characteristics can be explained by the SCLC model^59–62^.

**Figure 5:**
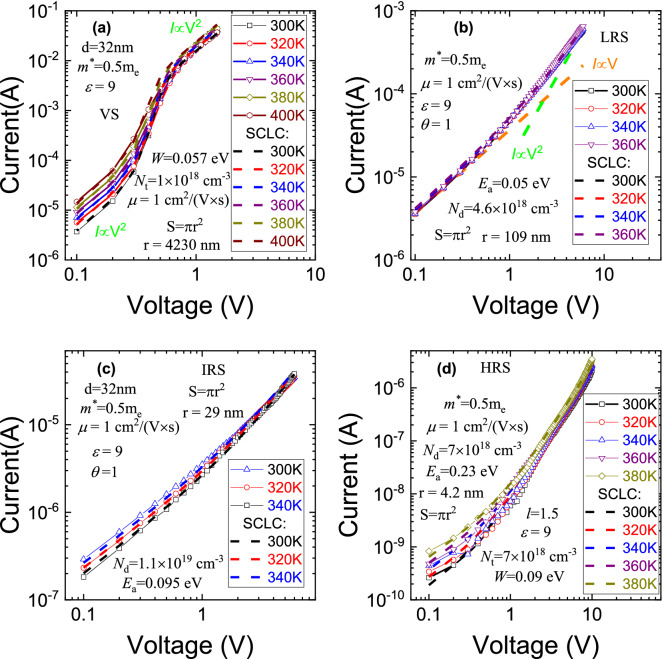
*I–V* characteristics and of p^+^-Si/SiN_x_/Ni and simulation by SCLC model in (**a**) VS, (**b**) LRS, (**c**) IRS, (**d**) HRS.

The memristor *I*-*V* characteristics in the VS are presented in Fig. 5a on a double logarithmic scale. It is shown in the figure that there are 3 regions in the experimental data: quadratic region, transition region and, again, quadratic region. To describe the transport mechanism, the following empirical formula derived from SCLC^62^ was used:

1 $$I = S\frac{9}{8}\mu \varepsilon \varepsilon_{0} \theta \frac{{U^{2} }}{{d^{3} }}\tanh \left( {\frac{U - A}{B} + C} \right),$$

$$\theta = \frac{1}{{1 + \frac{{N_{t} }}{{N_{c} }}\exp \left( {\frac{{W_{t} }}{kT}} \right)}},\;N_{c} = 2\left( {\frac{{2\pi m^{*} kT}}{{h^{2} }}} \right)^{{{\raise0.7ex\hbox{$3$} \!\mathord{\left/ {\vphantom {3 2}}\right.\kern-\nulldelimiterspace} \!\lower0.7ex\hbox{$2$}}}} ,$$

Here *S*—average efficient conductive area, *μ*—electron mobility, *ε*—static dielectric constant, *ε*_*0*_—dielectric constant, *d*—dielectric thickness, θ—the fraction of free electrons from all injected (trapped and free), *A*, *B*, *C*—transition region empirical parameters for stitching two quadratic regions, *N*_*c*_—effective density of states, *E*_*a*_—donor activation energy, *k*—Boltzmann constant, *T*—temperature, *N*_*t*_—trap concentration, *W*_*t*_—trap energy, m^*^—electron effective mass and *h*—Planck constant.

There are many selection parameters in the SCLC model. To reduce their number, the effective mass was taken as m^*^ = 0.5 m_e_ and the filament mobility was taken as the amorphous silicon mobility *μ* = 1 cm^2^/(V sec). The static dielectric constant value of 9 for SiN_*x*_ was taken from the range 7 for Si_3_N_4_ and 12 for Si. The third quadratic region was used for obtaining other parameters. Comparing the experiment and the SCLC model, we obtained the parameters: *N*_*t*_ = 1 × 10^18^ cm^−3^, *W*_*t*_ = 0.057 eV and *r* = 4230 nm. In a real memristor, the filament radius is about 10–100 nm. The effective radius 4230 nm is a model approximation of many filaments that we assume to be present in the initial VS, due to similarity with the *I*-*V* characteristics in a SiO_*x*_-based memristor^18^. Different empirical *A*, *B*, *C* parameters were obtained for different temperatures (Table 2). To obtain a more accurate formula for the transition region, it is necessary to numerically solve the equation system of dimensionless current and voltage and 3 dimensionless parameters^62^.

**Table 2:** Transition region empirical parameters for SCLC model in VS.

	Temperature	A	B	C
VS	300 K	0.5	0.1	1.05
	320 K	0.5	0.1	1.06
	340 K	0.5	0.1	1.07
	360 K	0.5	0.1	1.085
	380 K	0.47	0.1	1.01
	400 K	0.44	0.1	1.012

The LRS is described by the SCLC^59–62^ model with ohmic and quadratic parts:

2 $$I = I_{{{\text{Ohm}}}} + I_{{{\text{SCLC}}}} = Se\mu n\frac{U}{d} + S\frac{9}{8}\mu \varepsilon \varepsilon_{0} \theta \frac{{U^{2} }}{{d^{3} }},$$

$$n = \frac{{2N_{d} }}{{1 + \sqrt {1 + \frac{{4gN_{d} }}{{N_{c} }}\exp \left( {\frac{{E_{a} }}{kT}} \right)} }},\;\theta = \frac{1}{{1 + \frac{{N_{t} }}{{N_{c} }}\exp \left( {\frac{{W_{t} }}{kT}} \right)}},\;N_{c} = 2\left( {\frac{{2\pi m^{*} kT}}{{h^{2} }}} \right)^{{{\raise0.7ex\hbox{$3$} \!\mathord{\left/ {\vphantom {3 2}}\right.\kern-\nulldelimiterspace} \!\lower0.7ex\hbox{$2$}}}} ,$$

Here *S*—average efficient filament cross-section area, *e*—electron charge; *μ*—electron mobility, *n*—free electron concentration in the dielectric, *d*—dielectric thickness, *ε*—static dielectric constant, *ε*_*0*_—dielectric constant, θ—the fraction of free electrons from all injected (trapped and free), *N*_*d*_—donor-like defect concentration, g—degeneracy factor, *N*_*c*_—effective density of states, *E*_*a*_—donor-like defect activation energy, *k*—Boltzmann constant, *T*—temperature, *N*_*t*_—trap concentration, *W*_*t*_—trap energy, m^*^—electron effective mass and *h*—Planck constant.

The current in the LRS has a weak temperature dependence (Fig. 5b). The *I–V* characteristic in the LRS is similar to the current behavior in the SCLC model with filled traps^59–62^. This means that parameter θ is equal to 1. Fitting the experimental *I-V* characteristics for the LRS by the SCLC model gives the following parameter values: the filament radius *r* is 109 nm, the donor-like defect ionization energy is *E*_a_ = 0.05 eV, donor-like defect concentration—*N*_d_ = 8∙1 cm^-3^ at other fixed parameters.

The current in the IRS has also a weak temperature dependence at a low voltage (Fig. 5c). This state is also described by the SCLC model with filled traps^59–62^. The conductive area radius *r* = 29 nm decreases further. The fitting parameter of the donor-like defect ionization energy was *E*_a_ = 0.095 eV, and the donor-like defect concentration was *N*_d_ = 1.1 × 10^19^ cm^−3^. We must take into account that the IRS in our SiN_*x*_-based memristor is not controllable and not very stable: that is, our memristor has intermediate states, but it is not possible to get to the one that we need.

In the HRS, the temperature dependence is the strongest of all states and the formula is applied for the filling traps (Fig. 5d). At high voltages, the curve slope is not proportional to the square, that is, you need to use the formula for the SCLC case of exponential trap distribution^60,69^:

3 $$I = Se\mu n\frac{U}{d} + S\frac{9}{8}\mu \varepsilon \varepsilon_{0} \theta \frac{{U^{2} }}{{d^{3} }} + SN_{c} \mu e^{1 - l} \left( {\frac{\varepsilon l}{{N_{c} \left( {l + 1} \right)}}} \right)^{l} \left( {\frac{2l + 1}{{l + 1}}} \right)^{l + 1} \frac{{U^{l + 1} }}{{d^{2l + 1} }},$$

$$n = \frac{{2N_{d} }}{{1 + \sqrt {1 + \frac{{4gN_{d} }}{{N_{c} }}\exp \left( {\frac{{E_{a} }}{kT}} \right)} }},\;\theta = \frac{1}{{1 + \frac{{N_{t} }}{{N_{c} }}\exp \left( {\frac{{W_{t} }}{kT}} \right)}},\;N_{c} = 2\left( {\frac{{2\pi m^{*} kT}}{{h^{2} }}} \right)^{{{\raise0.7ex\hbox{$3$} \!\mathord{\left/ {\vphantom {3 2}}\right.\kern-\nulldelimiterspace} \!\lower0.7ex\hbox{$2$}}}} ,$$

where *l* = *T*_t_/*T*, *T*_t_ is the temperature parameter that characterizes the exponential trap distribution. From the curve slope, we get *U* at the degree equal to 2.5. Therefore, *l* is equal to 1.5. The simulation showed that, when switching to this state, the filament radius is decreased to 4.2 nm. The donor-like defect concentration was 7 × 10^18^ cm^−3^ and their activation energy was 0.23 eV. In the HRS state, the SCLC current flows in the trap-mediated mode with the trap concentration of *N*_t_ = 7 × 10^18^ cm^−3^ and the (trap ionization energy) depth of 0.09 eV.

## Discussion

We found that the Frenkel effect model of Coulomb isolated trap ionization, Hill-Adachi model of overlapping Coulomb potentials, Makram-Ebeid and Lannoo model of multiphonon isolated trap ionization, Nasyrov–Gritsenko model of phonon-assisted tunneling between traps, Shklovskii–Efros percolation model, Schottky model and the TAT mechanisms do not describe the charge transport in the SiN_*x*_-based memristor due to the fitting parameter values are unphysical or do not correspond to the SiN_*x*_ material.

All resistance states are described by the SCLC model; switching to other resistances is explained by a decrease in the conducting filament area. In the VS state, the filament radius is *r* = 4230 nm. We assume that a large effective filament area with such radius is explained by the presence of a larger filament number. When switching in the LRS state, the conductive region is decreased (*r* = 109 nm), that is, most of the filaments break or dissolve and, in the future, only a few major filaments are to be involved in the transport. Since we assume in the simulation that only the main filament is involved in a further switching and, during the switching, the area changes around it, in order to return to the initial resistance from the HRS, it is necessary to apply a voltage much higher than the operating switching voltage, which leads to an irreversible breakdown of the structure. The donor-like defect concentration sets the slope of the theoretical *I*–*V* curve in the ohmic part and the donor-like defect activation energy sets the current temperature dependence. The trap concentration and trap ionization energy give the curve slope and current temperature dependence in the SCLC part.

The following assumptions were made to simulate all states in the SCLC model. The conductive filament thickness does not change, although, in a real memristor, especially in the HRS, the conductive channel is dissolved partially or completely. In addition, we did not take into account the possibility of structural changes in the conductive channel during the resistance switching.

## Conclusion

In conclusion, two contact-limited and five bulk-limited models were applied to the simulation of the experimental charge transport of the forming-free SiN_*x*_-based memristor fabricated by the PECVD method in various states. The Schottky model, thermally assisted tunneling model, Frenkel model of isolated Coulombic trap ionization, Hill–Adachi model of overlapping Coulombic centers, Makram-Ebeid and Lannoo multiphonon isolated trap ionization model, Nasyrov-Gritsenko model of phonon-assisted electron tunneling between nearby traps and Shklovskii–Efros percolation model do not describe the charge transport in the SiN_*x*_-based memristor. The experimental *I*–*V* characteristics of the forming-free SiN_*x*_-based memristor fabricated by the PECVD method in the initial, high-resistance, intermediate and low-resistance states are quantitatively described by the space charge limited current model. The empirical SCLC formula was used to describe the transition region in the initial state, but the trap parameters were obtained from the quadratic SCLC model. The charge transport in the intermediate and low-resistance states of the SiN_*x*_-based memristor is described by the SCLC model with filled traps. The charge transport in the high-resistance state is described by SCLC with the exponential trap distribution. The decrease in the conductive region is explained by the resistance switching from the initial state to the high-resistance state.

## Methods

Non-stoichiometric silicon nitrides (a-SiN_*x*_:H) were obtained by plasma-enhanced chemical vapor deposition (PECVD) from a SiH_4_-N_2_ gas mixture under a controlled gas flow. Homogeneous a-SiN_*x*_:H films were deposited onto p^++^-type Si wafers purified from natural oxide at the PECVD reactor with a wide-aperture source and inductive excitation (at excitation frequency 13.56 MHz). The residual pressure in the working chamber was less than 10^–6^ torr, and it was reached by using a turbomolecular pump. The monosilane flow (gas mixture of 10% SiH_4_ diluted with Ar) supplied to the reaction zone was constant and amounted 10 cm^3^/min. The a-SiN_*x*_:H films of various compositions were obtained by changing the N_2_ flow rate in the range from 4 to 10 cm^3^/min and the generator high-frequency power of 200 W. The substrate temperature was maintained at 200 °C.

To study the memristor properties, a-SiN_*x*_:H films with a thickness of ~ 33 nm were grown. The upper nickel electrodes with a thickness of ~ 200 nm and with the area of ~ 0.5 mm^2^ were deposited through a metal mask by the magnetron sputtering in the Ar atmosphere. To improve the bottom electrode contact, a continuous nickel layer of the same thickness was deposited on the heavily doped substrate backside.

With the aim of high-spatial scanning ellipsometer Microscan-3 M (ISP SB RAS)^70^ central region (12 × 15 mm^2^), the sample was mapped. The mapping steps (x, y) were: 0.2 mm, and the light beam (hν = 1.96 eV) angle on the sample was 60°. The laser beam was focalized into a 10 μm light spot with a high-quality non-polarizing microlens. The ellipsometer is equipped with the computer-operated scanning stage that allows measuring optical parameters distribution over a sample surface up to 150 × 150 mm^2^. A four-zone measurement technique was used followed by averaging over all zones^24,70^. The thickness in each scanning point of the SiN_*x*_ layer was calculated independently by solving the numerical-inversion problem of ellipsometry for the simple optical model^24^: Si—SiN_*x*_ (*n* = 1.689).

To compare the experimental data and theoretical model, the simulation method of least absolute deviations (LAD) was used^71^. It consists of the theoretical model parameter selection process until the maximum deviation value (20%) of the theory from the experiment is reached. With this simulation method at Δ_max_ < 20%, we have the following accuracy of parameters: *r* = *r* ± 2 nm, μ = μ ± 0.02 cm^2^/(V s), *E*_*a*_ = *E*_*a*_ ± 0.01, *W*_*t*_ = *W*_*t*_ ± 0.01, *N*_*d*_ = *N*_*d*_ ± 0.1, *N*_*t*_ = *N*_*t*_ ± 0.1, m* = m* ± 0.02.

The voltage ramp-rate used for the *I–V* measurement was 0.9 V/sec. The endurance of the SiN_*x*_-based memristor at room temperature was measured with these parameters: Vread = − 1 V, Pulse width = 5 ms, Vset =  + 18 V, Vreset = − 19 V.

To measure the retention, the memristor was switched to the measured state at a temperature of 85 °C, and, every 0.1 s, a measurement was carried out at constant voltage − 1 V for about 4400 s. The experimental data approximation on the retention in the LRS and HRS was carried out from 4400 s to 10 years using the tangent of the experimental data. This approximation to 10 years is rough and can contain up to 30% errors. The supplementary material contain a detailed description and formulas of other charge transport models which used in this paper.

## Supplementary Information

Supplementary Information.
