# Supplementary material for: Charge transport mechanism in the forming-free memristor based on silicon nitride
Source: Sci Rep. 2021 Jan 28;11:2417. doi: 10.1038/s41598-021-82159-7 (PMC7843651; doi:10.1038/s41598-021-82159-7)
Supplement: Supplementary file 1 — Supplementary Information. [file 41598_2021_82159_MOESM1_ESM.docx]

Charge transport mechanism in the forming-free memristor based on silicon nitride

Andrei A. Gismatulin^1,2^, Gennadiy N. Kamaev^1^, Vladimir N. Kruchinin ^1^, Vladimir A. Gritsenko^1,2,3^, Oleg M. Orlov^4,5*^ and Albert Chin^6^

^1^Rzhanov Institute of Semiconductor Physics. Siberian Branch, Russian Academy of Sciences, Novosibirsk, Russia

^2^Novosibirsk State University,2 Pirogov street, Novosobirsk, 630090, Russia

^3^Novosibirsk State Technical University, 20 K. Marx ave., Novosibirsk, 630073, Russia

^4^Molecular Electronics Research Institute, 1st Zapadnyi Proezd 12/1, 124460, Zelenograd, Moscow, Russia

^5^Moscow Institute of Physics and Technology, 9 Institutskiy per, 141701, Dolgoprudny, Moscow Region, Russia

^6^Department of Electronics Engineering, National Chiao Tung University, Hsinchu 300, Taiwan

Correspondence and requests for materials should be addressed to Oleg Orlov (email: oorlov@niime.ru, phone number: +7(953)791-81-84)

**Supplementary data**

In the Schottky effect, the current is limited by the thermal electron emission through the barrier, which is reduced due to image forces. The Schottky effect current is described by equation^48^:

$I=AST^{2}\exp\left(-\frac{W_{0}-\left(\frac{e^{3}}{4\pi\varepsilon_{\infty}\varepsilon_{0}}\right)^{1/2}\sqrt{\frac{U}{d}}}{kT}\right),$ (1)

$A=\frac{4\pi m^{*}k^{2}e}{m_{e}h^{3}}=120.4\frac{m^{*}}{m_{e}}\left[\frac{A}{cm^{2}K^{2}}\right]$ (2)

Here *I* – current, *A* – Richardson-Deshman constant, *S* – contact area, *T* – temperature, *W_0_* – potential barrier height at the Ni/SiN*_x_* interface, *U* – voltage, *d* – dielectric thickness, *k* – Boltzmann constant, *e* – electron charge, *ε_∞_* = *n*^2^ – high frequency dielectric permittivity, *n* – refractive index, *ε_0_* – dielectric constant, *m^*^* – electron effective mass, *m_e_* – electron mass, *h* – Plank constant.

The thermally assisted tunneling (TAT) model is described the charge transport mechanism where electron after phonon absorption excited to certain energy and then tunnel through a triangle barrier. The current for the TAT model has the formula ^49,50^:

$I=\sqrt{2\pi m^{*}kT}\left(\frac{e}{h}\right)^{2}S\frac{U}{d}\exp\left(-\frac{W_{0}-\frac{1}{6}\left(\frac{he\times\left(\frac{U}{d}\right)}{4\pi kT\sqrt{m^{*}}}\right)^{2}}{kT}\right),$ (3)

The current *I* through the material containing traps can be described by the equation:

$I=eN^{2/3}SP$ (4)

Here *N* = *a*^-3^ – trap concentration, *a* – average distance between traps, *S* – contact area and *P* – trap ionization probability.

The Frenkel effect is due to a lower Coulomb positively charged trap potential in a strong electric field ^51,52^. The Coulomb trap ionization probability has the form:

$P=\nu\exp\left(-\frac{W-\left(\frac{e^{3}}{\pi\varepsilon_{\infty}\varepsilon_{0}}\right)^{1/2}\sqrt{\frac{U}{d}}}{kT}\right),$ (5)

Here *ν=W/h* – attempt to escape factor, *W* –trap ionization energy.

In the Hill-Adachi (H-A) model of overlapping Coulomb traps the trap ionization probability is described by equation ^53,54^:

$P=2\nu\exp\left(-\frac{W-\frac{e^{2}}{\pi\varepsilon_{\infty}\varepsilon_{0}s}}{kT}\right)\sinh\left(\frac{e\frac{U}{d}s}{2kT}\right)$ (6)

According to the Makram-Ebeid and Lannoo (ME-L) model, the charge transport in a dielectric is controlled by the multiphonon isolated trap ionization ^55^. The current density is described by equation (4) and the trap ionization probability in the ME-L model is described by equations (7, 8):

$P=\sum\exp\left(\frac{nW_{ph}}{2kT}-\frac{W_{opt}-W_{t}}{W_{ph}}\coth\frac{nW_{ph}}{2kT}\right)I_{n}\left(\frac{W_{opt}-W_{t}}{W_{ph}\sinh\left(W_{ph}/2kT\right)}\right)P_{i}\left(W_{t}+nW_{ph}\right)$ (7)

 (8)

Here *W*_t_ – thermal trap energy, *W*_opt_ – optical trap energy *W_ph_* – phonon energy, *I_n_* – modified Bessel function, *P_i_* – tunneling probability through a triangular barrier, *ћ* = *h*/2π – Plank constant.

The electron is excited from the ground state with the multiphonon absorption and it is tunnels to a neighboring trap due to a large integral overlapping value in the Nasyrov-Gritsenko (N-G) model ^56^. In the N-G model, the trap ionization probability is given by the equation:

 (9)

The charge transport in a non-stoichiometric dielectric can be described according to the Shklovskii-Efros (S-E) percolation model ^57,58^. This model assumes that excited electrons with an energy higher than the percolation energy *W_e_* are delocalized and, driving round a random potential, they transfer the charge. The current-voltage characteristics have the form:

$I=I_{0}\exp\left(-\frac{W_{e}-\text{(}Ce\frac{U}{d}aV_{0}^{\gamma}\text{)}^{\frac{1}{1+\gamma}}}{kT}\right),$ (10)

Here *I*_0_ is the preexponential factor, *W_e_* is the percolation energy, *a* is the space scale of fluctuations, *V*_0_ is the energy fluctuation amplitude, *C*≈0.25 is a numeric constant, *ɣ* is the critical index and it is equal to 0.9.

Due to the low temperature dependence, VS, IRS and LRS were simulated by the tunnel carrier injection at the contact by the Fowler-Nordheim mechanism ^63^:

$I=S\frac{e^{3}}{8\pi hW_{0}}\left(\frac{U}{d}\right)^{2}\exp\left(-\frac{\frac{8\pi\sqrt{2}}{3he}W_{0}^{\frac{3}{2}}\sqrt{m*}}{\frac{U}{d}}\right),$ (11)

Here *W_0_* – potential barrier height at the Ni/SiN*_x_* interface. Fowler-Nordheim.
